# Accuracy Analysis of the Multiparametric Acoustic Voice Indices, the VWI, AVQI, ABI, and DSI Measures, in Differentiating between Normal and Dysphonic Voices

**DOI:** 10.3390/jcm13010099

**Published:** 2023-12-23

**Authors:** Virgilijus Uloza, Kipras Pribuišis, Nora Ulozaite-Staniene, Tadas Petrauskas, Robertas Damaševičius, Rytis Maskeliūnas

**Affiliations:** 1 Department of Otorhinolaryngology, Lithuanian University of Health Sciences, 50061 Kaunas, Lithuania; virgilijus.ulozas@lsmuni.lt (V.U.); kipras.pribuisis@lsmuni.lt (K.P.); tadas@petrauskas.co.uk (T.P.); 2 Faculty of Informatics, Kaunas University of Technology, 51368 Kaunas, Lithuania

**Keywords:** acoustic voice analysis, screening, DSI, AVQI, ABI, VWI

## Abstract

The study aimed to investigate and compare the accuracy and robustness of the multiparametric acoustic voice indices (MAVIs), namely the Dysphonia Severity Index (DSI), Acoustic Voice Quality Index (AVQI), Acoustic Breathiness Index (ABI), and Voice Wellness Index (VWI) measures in differentiating normal and dysphonic voices. The study group consisted of 129 adult individuals including 49 with normal voices and 80 patients with pathological voices. The diagnostic accuracy of the investigated MAVI in differentiating between normal and pathological voices was assessed using receiver operating characteristics (ROC). Moderate to strong positive linear correlations were observed between different MAVIs. The ROC statistical analysis revealed that all used measurements manifested in a high level of accuracy (area under the curve (AUC) of 0.80 and greater) and an acceptable level of sensitivity and specificity in discriminating between normal and pathological voices. However, with AUC 0.99, the VWI demonstrated the highest diagnostic accuracy. The highest Youden index equaled 0.93, revealing that a VWI cut-off of 4.45 corresponds with highly acceptable sensitivity (97.50%) and specificity (95.92%). In conclusion, the VWI was found to be beneficial in describing differences in voice quality status and discriminating between normal and dysphonic voices based on clinical diagnosis, i.e., dysphonia type, implying the VWI’s reliable voice screening potential.

## 1. Introduction

A multidimensional approach is used in clinical practice to diagnose laryngeal/voice abnormalities. This approach includes subjective evaluation of a voice both by the medical professional and the patient, objective measurement of voice acoustics and voice aerodynamics, and visualizing the larynx using video laryngostroboscopy (VLS) [1]. 

In this context, acoustic voice analysis plays a crucial role in the assessment of vocal function and diagnostics in phoniatrics and laryngology [2]. Voice acoustic data are noninvasive, reasonably easy-to-capture, and accurate biomarkers that also offer workable and trustworthy options for dysphonia screening and monitoring. Therefore, measurement of acoustic voice signals represents the most commonly used instrumental tool in clinical practice and research for objectively and quantitative characterizing voice quality [3,4].

In the last decades, numerous acoustic analysis algorithms were developed to measure the pitch, amplitude and waveform perturbation, and spectral and cepstral characteristics of sound waves [2,5]. In order to address the limiting validity of a single acoustic parameter in comparison to the multidimensionality of voice signals, researchers have created several multiparametric acoustic voice indices (MAVIs) during the past few decades. These indices assess and fuse multiple acoustic voice parameters based on the domains of time, frequency, amplitude, and quefrency while taking into consideration both sustained phonation and connected speech and provide a single score that measures voice quality [6,7,8]. 

Nowadays, several MAVI models based on sustained vowels and continuous speech have been introduced in research and clinical practice for the evaluation of voice quality: the Dysphonia Severity Index (DSI), the Acoustic Voice Quality Index (AVQI), the Acoustic Breathiness Index (ABI), and the Voice Wellness Index (VWI). 

Wuyts et al.’s DSI model, presented in 2000, is a multivariate model that provides an objective and quantitative indicator of overall voice quality by incorporating acoustic (jitter, and the lowest intensity and highest fundamental frequency in the vocal range profile) and aerodynamic (maximum phonation time of the vowel [a:]) markers [9]. DSI has been regarded as a valuable and viable assessment for assessing overall voice quality, voice treatment, vocal training, and phonosurgery results [10,11,12,13,14,15,16,17]. Additional research found connections between the DSI and auditory-perceptual judgment and quality of life evaluation, establishing the DSI as a valid approach for evaluating dysphonia severity [13,14,15,18,19,20]. The findings of the comparison research revealed that the DSI and AVQI’s performances were comparable with an elevated degree of accuracy in distinguishing among normal and dysphonic voices [21].

The DSI is originally scored from −5 to +5, in which an average subject with a normal healthy voice has a score of +5, and −5 indicates a severely disordered voice [9]. However, it should be noticed that the DSI value might vary across different geographic regions, age, vocal performance, and ethnic groups [19,22,23,24]. In meta-analysis performed on a group of healthy adult participants, the mean normative value of the DSI was +3.05 (the confidence level was 2.13–3.98) [25]. 

The AVQI is a six-variable acoustic model developed by Maryn et al. in 2010 [26] for the multiparametric measurement of voice quality concatenating both the sustained vowel [a:] and the voiced parts of a continuous speech fragment. The equation of the AVQI includes acoustic markers from time, frequency, and quefrency domains, and it is a multidimensional representation of the dysphonia severity. The AVQI scores may range from 0 to 10 points with a higher score indicating more severe dysphonia. Numerous studies have confirmed the remarkable features of the AVQI, including its high consistency, concurrent validity, test-retest reliability, high sensitivity to changes in voice quality brought about by voice therapy, usefulness in differentiating between dysphonia severity levels perceptually, and adequate diagnostic accuracy between normal and pathological voices with good discriminatory power [27,28,29,30]. The AVQI values are independent of age and gender, which expands the possibilities for the further generalization of this tool for potential voice-screening applications [24]. In consequence, the AVQI is currently regarded as a globally recognized multiparametric voice quality assessment instrument for clinical and research applications [31,32,33]. 

The ABI is a multiparametric, nine-variable acoustic measure based on concatenated samples of continuous speech and the sustained vowel /a/ to quantify the degree of breathiness with a single score, and was developed by Barsties v Latoszek in 2017 [34]. The ABI score ranges from 0 to 10, and the higher an ABI score, the more severe the breathiness, and vice versa.

The ABI revealed highly reliable results in a test-retest measurement of vocally healthy subjects [35]. The results of several studies confirmed the ABI as a robust and valid objective measure for evaluating breathiness because ABI scores and perceived breathiness ratings were shown to be strongly correlated; however, neither age and gender nor roughness significantly affected the ABI in the evaluation of natural voices [4,36]. In addition, the ABI also indicates highly sensitive therapy-related voice quality changes and, therefore, is useful for therapy studies in order to more accurately characterize differences in voice quality before and after treatment [4,37]. Also, the ABI appears to be relatively robust to phonetic inter-language differences [38]. The diagnostic accuracy of the ABI in distinguishing between normal and pathological voices revealed in different validation studies showed high to very high results in terms of both sensitivity and specificity [37]. 

The VWI integrates the voice-related data from two different information sources (i.e., acoustic voice analysis, such as the AVQI and Glottal Function Questionnaire (GFI), as patient-reported outcome measures) and supports the concept that the voice assessment process should consider the multidimensionality involved in the manifestation of voice disorders. The VWI is the equalizing proportion summation of the AVQI and GFI scores [39]. The VWI scores may range from 0 to 20 points with a higher score indicating more severe dysphonia. The results of the recent study showed that VWI application represents an accurate and reliable tool for voice quality measurement and normal versus pathological voice screening, manifesting in excellent diagnostic accuracy (AUC = 0.972) and the best balance between sensitivity (94.15%) and specificity (95.72%) [39]. 

The GFI questionnaire was developed by Bach et al. in 2005 [40]. It can be used as a compounding part of the VWI and represents a concise (four-item) and reliable symptom-based self-administered tool, which is focused on the functional aspects of voice disorder and easily comprehensible. Its purpose is to assess the extent of vocal dysfunction in adults. The GFI scores may range from 0 to 20 points with a higher score indicating more severe vocal dysfunction. The later studies revealed the GFI cut-off score of >3.0 points distinguishing dysphonic patients from healthy normal voice controls with a high level of sensitivity and specificity [41]. Additionally, the dysphonia screening potential of GFI was revealed by merging separate acoustic voice parameters with responses to GFI questions and combining AVQI and GFI measurements [42].

The examination of comparison research data indicated equal findings for the DSI and AVQI in terms of identifying normal and dysphonic voice, although the AVQI had greater validity features. Based on auditory-perceptual judgment, the research team concluded that the AVQI appears to be useful in defining variations in vocal quality state and distinguishing between normal and dysphonic voices [21]. However, the consequent study yielded that both these MAVIs can also differentiate between vocally healthy and voice-disordered subjects in comparison with the dysphonia classification based on the diagnosis of laryngeal disorder, thus enabling the quantification of abnormality [43]. In 2023, Penido et al. evaluated the AVQI, ABI, and DSI for speech–language pathologist decision-making in the assessment of teachers’ voice complications. The findings of their study revealed that the AVQI, ABI, and DSI are measures that may provide substantial voice information and assist vocal healthcare providers in deciding on whether instructors should be professionally limited in their vocal activities [30]. 

However, the comparison of the MAVI in respect to the voice screening problem has not been tested before. Therefore, the aim of the study was to investigate and compare the accuracy and robustness of the multiparametric acoustic voice indices, the VWI, AVQI, ABI, and DSI measures in differentiating between normal and dysphonic voices.

## 2. Materials and Methods

The examinations of study participants took place at the Department of Otolaryngology, Lithuanian University of Health Sciences, Kaunas, Lithuania. All data from individuals with voice disorders were collected before any treatment, constituting the baseline. Informed consent was obtained from all the participants before their involvement in the study.

The inclusion criteria for the normal voice subgroup were as follows: (a) self-perceived normal voice with no voiced-related complaints, (b) absence of chronic laryngeal diseases or voice disorders history, (c) absence of pathological laryngeal alterations based on video videolaryngostroboscopy (VLS), and (d) evaluation of voice samples as normal by a laryngologist.

The pathological voice subgroup included a variety of laryngeal diseases and voice disturbances, notably benign and malignant mass lesions of the vocal folds and unilateral vocal fold paralysis. The inclusion criteria for this subgroup were: (a) complaints of voice disorders, (b) voice assessed as pathological by a laryngologist, (c) presence of laryngoscopically positive signs, and (d) histologically verified diagnosis in cases of mass lesions of the vocal folds.

The diagnosis of voice disorders relied on clinical examination (complaints and history), VLS, and histological verification of excised mass lesions of the vocal folds. Positive laryngoscopic findings comprised vocal fold hypertrophy, paralysis, and benign and malignant mass lesions of the vocal folds. Endolaryngeal microsurgical interventions were performed on subjects with mass lesions, and the diagnosis was verified by histological evaluation of the excised tissue. The final diagnosis was used to assess the diagnostic accuracy of the investigated MAVI in distinguishing among normal and pathological voice participants.

### 2.1. Glottal Function Index Questionnaire

Each participant of the study (normal and pathological voice subgroups) filled in the GFI questionnaire at the baseline, i.e., pre-treatment, along with voice recordings. 

### 2.2. Voice Recordings

Voice recordings from the research participants were collected using a studio oral cardioid AKG Perception 220 microphone (AKG Acoustics, Vienna, Austria) in a T-series soundproof room for auditory assessment (T-room, CATegner AB, Bromma, Sweden). The microphone was set 10.0 cm away from the lips, maintaining a 90° microphone-to-mouth angle. Every individual was assigned two voice tasks that were recorded digitally. The challenges included phonating the vowel sound [a:] for at least 4 s and reciting a phonetically balanced text fragment in Lithuanian “Turėjo senelė žilą oželį” (“The granny had a small grey goat”). The respondents were told to execute both voice activities at their personal volume and pitch. These narrations were recorded using the Audacity audio recording application (https://www.audacityteam.org/, accessed on 11 October 2023), at a sampling rate of 44.1 kHz and saved for storage on the computer’s hard disk drive in a 16-bit resolution uncompressed “wav” audio file format.

### 2.3. DSI Estimation

The DSI was calculated using the Voice Diagnostic Center (VDS) (lingWAVES software, version 2.5, WEVOSYS, Forchheim, Germany). Firstly, the jitter percentage was calculated using a sustained vowel [a:] of no less than 2 s. Secondly, following maximal inhalation, maximal phonation duration was determined for vowel [a:] sustained for as long as feasible at a usual pitch and loudness. Thirdly, the individuals’ voice range profiles were established. Only the lowest intensity (Ilow) and highest frequency (Fhigh) of the vocal range profiles were used to calculate the DSI. Lastly, the DSI was determined using lingWaves VDC Vospector analysis depending on the weighted combination of the highest frequency in Hz (FoHigh), lowest intensity in dBA (I-low), maximum phonation time in seconds (MPT), and jitter percentage.

### 2.4. AVQI Estimation

The Praat application (version 5.3.57; https://www.fon.hum.uva.nl/praat/, accessed on 11 October 2023) was used for processing the speech recordings for AVQI estimations. The speech recordings were combined in the following sequence: text segment, 2 s pause, 3 s sustained vowel/a/segment. The AVQI script version 02.02 designed for the Praat application was utilized for the acoustic analysis https://www.vvl.be/documenten-en-paginas/praat-script-avqi-v0203?download=AcousticVoiceQualityIndexv.02.03.txt, accessed 11 October 2023 [6]. 

### 2.5. ABI Estimation

For ABI calculations, the signal processing of the voice samples was conducted using the Praat software (version 5.4.22; https://www.fon.hum.uva.nl/praat/, accessed on 11 October 2023). The voice samples were analyzed using the ABI script developed for the Praat program (version 5.4.22): https://www.jvoice.org/cms/10.1016/j.jvoice.2016.11.017/attachment/c156729a-af1a-4973-b77d-940ccb085145/mmc1.docx, accessed on 11 October 2023 [4].

### 2.6. VWI Estimation

The “Voice Wellness Index” application for use both with iOS and Android operating devices was utilized for WVI estimation [39]. This application allows voice recording, automatically extracting acoustic voice features consisting of the AVQI, the GFI measures, and displaying the VWI result alongside a recommendation to the user.

### 2.7. Statistical Analysis

The statistical analysis was conducted using IBM SPSS Statistics for Windows, version 28.0.1.1 (Armonk, NY, USA: IBM Corp.) and MedCalc Version 20.118 (Ostend, Belgium, BE: MedCalc Software Ltd.). The chosen level of statistical significance was set at 0.05.

To assess the data distribution, the normality law was examined using the Shapiro–Wilk test of normality, along with the calculation of coefficients of skewness and kurtosis. In cases of normally distributed data, a Student’s *t*-test was employed to test the equality of means. An analysis of variance (ANOVA) was utilized to ascertain significant differences among the multiple means of independent groups [44].

The linear relationship between variables obtained from continuous scales was evaluated using Pearson’s correlation coefficient. To evaluate optimum sensitivity and specificity at appropriate cut-off values, receiver operating characteristic (ROC) curves were constructed. To assess discriminatory accuracy, the “area under the ROC curve” (AUC) was used. An AUC of more than 0.90 was deemed excellent, an AUC of less than 0.70 was considered low, and an AUC of less than 0.50 showed chance-level accuracy for diagnosis.

A pairwise analysis, as reported by De Long et al., was used to determine whether there were statistically significant variations among two or more factors when defining normal/pathological voices [45].

## 3. Results

### 3.1. Study Group

The research cohort comprised 129 adults, with 58 men and 71 women. The average age of the participants was 42.32 years (SD 14.83). Within the study, a subgroup of normal voices comprised 49 healthy volunteers (16 men and 33 women) with an average age of 31.69 years (SD 9.89). Conversely, the pathological voice subgroup consisted of 80 patients (42 men and 38 women) with an average age of 48.83 years (standard deviation 13.6). This subgroup presented a range of laryngeal diseases and associated voice disruptions, including benign and malignant mass lesions of the vocal folds and unilateral paralysis of the vocal folds.

The demographic data of the study group and diagnoses of the pathological voice subgroup are presented in Table 1. 

Findings from prior research indicated no significant correlations between the subjects’ age, sex, AVQI, and ABI measurements [31,36]. However, DSI values were found to be unrelated to sex but showed a slight correlation with age [43]. Consequently, in the current study, the control and patient groups were deemed appropriate for analyzing the investigated MAVI data, even though these groups were not matched in terms of sex and age.

### 3.2. MAVI Evaluation Outcomes

The statistical analysis of the mean MAVI scores demonstrated significant differences (*p* = 0.001) between the normal and pathological voice groups. The specific details regarding the mean scores for various MAVIs are presented in Table 2.

Table 2 demonstrates the separate MAVI scores for the normal and pathological voice groups. The findings indicate that the normal voice group exhibited statistically significantly lower mean scores when compared to the pathological voice group.

Moderate to strong positive linear correlations were observed between different MAVIs. Pearson’s correlation coefficients ranged from 0.446 to 0.881 and can be observed in Table 3. 

### 3.3. Normal vs. Pathological Voice Diagnostic Accuracy of the Investigated MAVI

**Figure 1:**
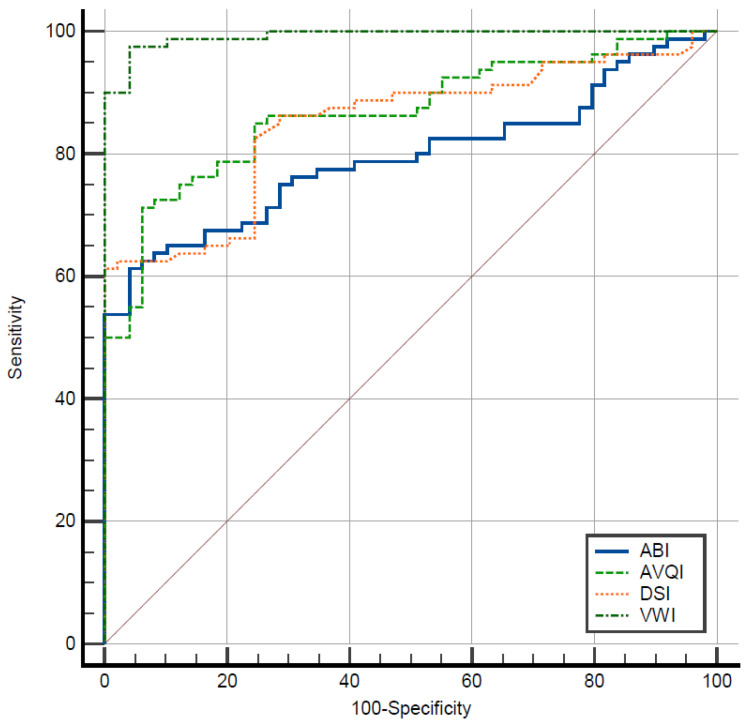
ROC curves illustrating the diagnostic accuracy of the Acoustic Breathiness Index (ABI), Acoustic Voice Quality Index (AVQI), Dysphonia Severity Index (DSI), and Voice Wellness Index (VWI) in discriminating between normal/pathological voices.

The ROC analysis was employed to assess the diagnostic accuracy of the investigated MAVI in distinguishing between normal and pathological voices. The ROC curves were visually examined to identify the optimal cut-off scores based on general interpretation guidelines [46]. Figure 1 displays the ROC curves for reference.

As depicted in Figure 1, the ROC curves generated from various MAVI values predominantly occupy the upper portion of the graph, surpassing the middle reference line. This observation distinctly underscores the commendable capability of the investigated MAVI in effectively distinguishing between normal and pathological voices. Notably, the VWI scores exhibited the largest area under the curve, indicating a higher predictive value and greater accuracy of this index in discerning between the normal and pathological voice groups. 

The results of the detailed comparative ROC statistical analysis and the descriptive outcomes of the MAVI between normal and pathological voice groups are presented in Table 4.

Table 4 provides an overview of the statistics concerning the MAVI’s ability to effectively differentiate between normal and pathological voice groups, yielding the following outcomes. The ROC statistical analysis indicated that all employed measurements exhibited a high accuracy (AUC of 0.80 and greater) and an acceptable balance of sensitivity and specificity in distinguishing between normal and pathological voices. The VWI, with an AUC of 0.99, demonstrated the highest diagnostic accuracy based on clinical diagnosis, specifically the dysphonia type. The highest Youden index, reaching 0.93, indicated a VWI cut-off of 4.45 corresponds to highly acceptable sensitivity (97.50%) and specificity (95.92%). Other MAVIs displayed AUCs ranging from 0.80 to 0.87, sensitivities from 61.25% to 71.25%, specificities from 95.92% to 100%, and Youden indices from 0.57 to 0.65, respectively. A further pairwise comparison of the AUC differences of separate MAVIs in discriminating between normal and pathological voices is presented in Table 5.

As demonstrated in Table 5, the pairwise comparison of the significance of the differences between the AUCs of separate MAVIs, as described by DeLong et al., revealed that considering the AUCs, the VWI showed the statistically significantly highest difference when compared to the other MAVIs used in this study. 

## 4. Discussion

For the very first time in a single research project and for exactly the same cohort of participants, the reliability of the multiparametric acoustic voice indices, the VWI, AVQI, ABI, and DSI measures in discriminating between normal and diseased voices was investigated in this study. Clinical evaluation, i.e., the findings of the examination of complaints, history, subjective voice assessment, laryngeal imaging, and histological research, was used to identify a *p* pathological voice. Strict standards for a normal voice were established. As a result, although diverse kinds of dysphonia were addressed, correct categorization between vocally healthy and voice-disordered participants was evaluated in the current investigation.

The results of this study, related to the ROC analysis, indicated that all four investigated indices, the VWI, AVQI, ABI, and DSI, revealed good discrimination between individuals with normal and pathological voices as determined via the clinical diagnosis of laryngeal disorder. However, among the four investigated indices, the VWI achieved an AUC of 0.99, sensitivity of 97.50%, and specificity of 95.92%, which showed greater power for reaching this goal. Thus, the comparative analysis of the results of the present study highlighted the significantly higher level of accuracy of the VWI in differentiating between normal and pathological voices, suggesting the reliable voice screening potential of the VWI. 

These outcomes, to some extent, can be considered as predictable and comprehensible. The current findings are consistent with the statement in the literature that amalgamating acoustic voice analysis and the results of a patient’s self-assessment provides complementary information that increases the strength, and reinforces the importance, of multidimensional assessment, thereby investigating different aspects of a voice disorder [33,42,47]. 

The results of the present study demonstrated the significantly higher power of the VWI obtained from voice recordings using a studio microphone to discriminate between normal and pathological voices compared to that of the DSI. The DSI is primarily regarded as an indicator of vocal function, and it is assumed to more accurately represent the capabilities or limits in vocal functioning, and it can be used as a universal measure of vocal performance and/or voice dysfunction [30]. The DSI includes just one acoustic parameter linked to voice quality (jitter percentage), and three other variables relating to voice performance and functionality: maximum phonation time, softest magnitude, and a higher frequency. The AVQI relies on six acoustic voice quality indicators and is regarded as being a superior indicator of overall voice quality [6], whereas the ABI relies on nine acoustic voice quality indicators and is better suited to identifying breathiness in voice quality, especially in cases of vocal fold nodules, paralysis, or paresis of the recurrent laryngeal nerve, and vocal fold bowing corresponding to presbyphonia [38]. 

In clinical practice, it is probable that people with or without laryngoscopic abnormalities cannot always be accurately classified by using auditory perceptual assessment or using acoustic parameters that have been validated as measures of perceived dysphonia severity. However, it is widely recognized from clinical experience that individuals exhibiting laryngoscopically aberrant symptoms can, in turn, produce a perceptually “normal” voice, and vice versa. This may be explained by the observation that the existence of a mass lesion or other structural variation in the vocal folds does not always result in dysphonia as perceived or as measured by acoustics, particularly if the lesion’s location has little bearing on the vocal folds’ vibratory characteristics. The VWI, which incorporates two sources of data known as the AVQI and GFI, guarantees that both of these modalities give related but distinct kinds of discriminating information useful for differentiating between healthy and pathological voices and boosts classification performance. 

It is important to note that, despite the relative ease and consistency of DSI registration, this technique necessitates the assistance of a professionally qualified speech therapist or phoniatrician. As a result, DSI estimation cannot be automated and completed as a vocal “self-assessment” by a person. As a result, despite a lengthy tradition of evaluating the overall quality of a voice based on sustained vowels, this DSI registration peculiarity reduces the DSI’s potential utility for voice pathology screening purposes. The multivariate structures of the VWI, AVQI, and ABI, on the other hand, depend on a linear regression model which incorporates pertinent acoustic parameters; they consist of both continuous speech and sustained vowel sounds in the acoustic evaluation, and the processing of signals employs freeware Praat algorithms, and can thus be standardized and made automated. This has already been realized in several applications available for AVQI estimation: VoiceEvalU8 [48], A Comprehensive Application for Grading Severity of Voice [29], VoiceScreen, version 4.4.22 [49], and ABI assessment: VOXplot, version 2.0 [50]. As a result, the registration of the AVQI, ABI, and VWI as an “ecologically valid” MAVI may be readily accomplished using specific programs, even without the presence of trained staff, allowing individuals to self-assess their voice quality. Consequently, these MAVIs suggest reliable voice screening options. Moreover, the VWI application provides recommendations to users based on the test results.

Merging the data from the two information sources has additional benefits for the VWI as the suitable method for differentiating between voice quality groups with and without disorders. The significant aspect of the VWI is its relatively high discrimination power based on the GFI data. Therefore, this sensor-independent data source with such a strong discrimination strength lessens the possibility of acoustic parameter-dependent variances resulting from variations in smartphone microphones and balances the effects of the two compounding parts (AVQI and GFI) on the VWI score. When using various voice recording devices, like various cellphones or other mobile communication devices, this capability is crucial. 

Several of the current study’s limitations must be taken into account. The study group of individuals with clinically discriminative organic laryngeal diseases and voice disorders served as the basis for the current study’s findings. In order to maximize the comparability of various MAVIs, more research is needed of a broad range of vocal disorders, including functional voice disorders. The voice recordings for the current investigation were made in a soundproof room. Nevertheless, in actual clinical settings with background noise, the omnidirectional inbuilt microphones in cellphones might produce different outcomes. Therefore, additional research is needed to assess how well the various MAVI applications work with various cellphones in a real-world clinical scenario, as well as the effects of the microphone’s peculiarities and the speech recording environment.

## 5. Conclusions

All MAVIs used in this study, namely the DSI, AVQI, ABI, and VWI, displayed good accuracy in distinguishing between normal and dysphonic voices. The VWI, on the other hand, yielded greater validity characteristics. As a result, the VWI appears to be useful in defining changes in voice quality status and distinguishing between normal and dysphonic voices based on clinical diagnosis, i.e., the dysphonia type, implying the VWI’s trustworthy voice screening capability.

## Figures and Tables

**Table 1:** Demographic data of the study group.

Diagnosis	n	Age	
		Mean	SD
Normal voice	49	31.69	9.89
Mass lesions of the vocal folds (vocal fold polyp, nodules, cyst, granuloma)	49	44.39	12.4
Vocal fold cancer (T1-2N0M0)	11	65.09	7.71
Chronic hyperplastic laryngitis	10	55.9	7.34
Unilateral vocal fold paralysis	6	40.83	12.77
Bilateral vocal fold paralysis	4	52.75	12.61
Total	129	42.32	14.83

Abbreviation: SD—standard deviation.

**Table 2:** Mean MAVI scores in normal and pathological voice groups.

MAVI	Voice Group	n	F	Mean	Std. Deviation	*p*
Acoustic Breathiness Index	Normal voice	49	18.59	3.28	1.17	0.01
	Pathological	80		5.33	2.08	
Dysphonia Severity Index	Normal	49	0.03	6.28	2.22	0.01
	Pathological	80		−0.49	5.83	
Acoustic Voice Quality Index	Normal	49	30.78	2.09	0.77	0.01
	Pathological	80		4.26	1.80	
Voice Wellness Index	Normal	49	35.41	2.53	1.14	0.01
	Pathological	80		9.29	3.01	

Abbreviations: MAVI—Multiparametric Acoustic Voice Index; F—degrees of freedom.

**Table 3:** Correlations between different MAVI scores.

MAVI	Acoustic Breathiness Index	Dysphonia Severity Index	Acoustic Voice Quality Index	Voice Wellness Index
Acoustic Breathiness Index	1	0.45 *	0.88 *	0.72 *
Dysphonia Severity Index	0.45 *	1	0.56 *	0.54 *
Acoustic Voice Quality Index	0.88 *	0.56 *	1	0.76 *
Voice Wellness Index	0.72 *	0.54 *	0.76 *	1

*—Correlations are significant at the 0.01 level (2-tailed), Abbreviation: MAVI—Multiparametric Acoustic Voice Index.

**Table 4:** ROC statistics illustrating the accuracy of the different MAVIs in differentiating between normal and pathological voices.

MAVI	AUC	Cut-off	Sensitivity %	Specificity %	Youden-Index J
Acoustic Breathiness Index	0.80	4.87	61.25	95.92	0.57
Dysphonia Severity Index	0.85	−4.3	61.25	100	0.61
Acoustic Voice Quality Index	0.87	3.27	71.25	93.88	0.65
Voice Wellness Index	0.99	4.45	97.50	95.92	0.93

Abbreviations: ROC—Receiver Operating Curve; MAVI—Multiparametric Acoustic Voice Index; AUC—area under the curve.

**Table 5:** A pairwise comparison of the AUC’s differences of separate MAVIs in discriminating between normal and pathological voices.

MAVI	Acoustic Breathiness Index	Dysphonia Severity Index	Acoustic Voice Quality Index	Voice Wellness Index
Acoustic Breathiness Index	-	0.053	0.073 *	0.198 *
Dysphonia Severity Index	0.053	-	0.02	0.145 *
Acoustic Voice Quality Index	0.073 *	0.02	-	0.125 *
Voice Wellness Index	0.198 *	0.145 *	0.125 *	-

*—Significance level *p* < 0.01 level, Abbreviation: MAVI—Multiparametric Acoustic Voice Index.

## Data Availability

The data presented in this study are available on request from the corresponding author.
